# Sample size calculations for pilot cluster-randomised controlled trial: a review 2010–2020

**DOI:** 10.1186/s40814-026-01792-z

**Published:** 2026-03-16

**Authors:** Rebecca M. Simpson, Jen Lewis, Michael J. Campbell, Lauren Desoysa, Peter J. Dodd, Dan Green, Bright C. Offorha, Ines Rombach, Stephen J. Walters, Steven Julious

**Affiliations:** 1 Sheffield Centre for Health and Related Research (SCHARR), University of Sheffield, Sheffield, UK; 2 College of Health and Life Sciences, Aston University, Birmingham, UK; 3 Division of Imaging, Informatics and Data Science, University of Manchester, Manchester, UK

**Keywords:** Pilot studies, Cluster trials, Sample sizes

## Abstract

**Background:**

There is no recommended guidance on appropriate sample sizes for pilot cluster-randomised controlled trials (cRCTs). Pilot trials should not aim to demonstrate efficacy, and achieving power should not be used to justify the sample size. However, the CONSORT extension for pilot trials states that some justification for their sample size should be given.

We conducted a review to understand the choices and justifications of pilot cRCT sample sizes and their trends over time, and to explore apparent changes following the publication of CONSORT extensions for cluster trials and for pilot and feasibility trials.

**Methods:**

We searched PubMed and Web of Science for pilot cRCTs. The search took place on 01/10/2020 and was restricted to papers published on or after 01/01/2010. Identification of papers was based around a search for the terms ‘pilot’ or ‘feasibility’ in the title and abstract/topic. The primary interest in the review was in the planned sample size in terms of clusters per-arm. We also examined participants per-arm and enrolled sample size. Analyses were descriptive or displayed graphically.

**Results:**

Our search returned 3090 records. After removing exact duplicates, aggregating records into unique studies and excluding ineligible studies, we identified 170 pilot or feasibility cRCTs. The median sample size was four clusters per arm. Stratification showed this to be fairly consistent, regardless of the justification given, whether a formal analysis was planned, whether the intention was to estimate the Intra-Cluster Correlation, cluster type, general medical area, funding type, and over time.

**Conclusion:**

Average sample sizes for cRCTs have remained strikingly constant over the period 2010–2020 and across several key features of studies; they do not appear to be meaningfully impacted by the stated study aims or sample size justifications. This is despite the fact that the reported main aims for pilot cRCTs, and justifications for their sample sizes, do appear to have changed during this time.

Given that aims and justifications appear to have changed, but typical sample sizes have not, it is possible that some researchers choose sample size for pilot cRCTs primarily out of convenience or practical reasons, whilst stating other justifications.

**Supplementary Information:**

The online version contains supplementary material available at 10.1186/s40814-026-01792-z.

## Introduction

Cluster-randomised controlled trials (cRCTs) are studies which randomise groups (‘clusters’) of patients or participants, rather than individuals. Example units of randomisation include general practices (GPs), hospitals, schools or geographical areas. The decision to undertake a cluster-randomised trial is often made for practical reasons such as ease of implementation, prevention of contamination across arms, or where the effect is expected at population level, e.g. with infectious diseases [1]. Some interventions, including health technology, may also be systems of care that would require a whole unit, such as a hospital, to be randomised. Within cRCTs, the outcomes of patients within clusters can be correlated, introducing an additional level of complexity to the design and analysis of the studies. This correlation is estimated by the intra-cluster correlation coefficient (ICC) and can occur for many reasons, including the common care or clinical practice of the patients within a cluster, where the cluster may be a GP or a clinician. Although the ICC may be small—often less than 0.03—it can have a major impact on the power of a study [2]. Due to required adjustments to the planned sample size to ensure sufficient power in a cRCT, they frequently have a much larger sample size than individually randomised trials.

For a given health technology or similar intervention, the feasibility of undertaking a cRCT can be investigated through a pilot study. The importance of pilot trials in health services research and the quality of their conduct and reporting is well understood [3–5], particularly where a main trial is anticipated to be large and expensive, which is more often the case for cRCTs. The publication of the 2012 cRCT extension to the CONSORT statement [6] reflects an increasing number of published cRCTs, and concomitantly there is an increased level of scrutiny on their conduct and reporting [7]. With reference to the current paper, this CONSORT extension draws attention to the importance of cRCT-specific issues around sample size, specifically, the ‘method of calculation, number of clusters (and whether equal or unequal cluster sizes are assumed), cluster size, a coefficient of intracluster correlation, and an indication of its uncertainty’. In addition, the 2016 pilot and feasibility trial extension to the CONSORT statement [8, 9] states that a rationale for the chosen pilot trial sample size should be given, and that it must be congruent with the study aims. However, despite the increased focus on both cluster randomisation and rigorous piloting, there is a lack of understanding as to what the sample size for a pilot/feasibility cRCT should be.

It is well accepted that pilot trials should not have the aim of demonstrating a significant effect [9–11]; as such, sample size should not be chosen in order to achieve statistical power. However, the CONSORT extension for pilot trials states that some justification for their size should be given [9]. There is, however, currently almost no guidance on appropriate sample sizes for pilot cRCTs. One source from 2009 suggests four clusters per arm as a minimum, citing the unsuitability of the two independent samples *t*-test for comparing outcomes in fewer clusters and the inability of the alternative non-parametric Wilcoxon/Mann–Whitney test to cope with fewer clusters [12]. However, since this recommendation is based around hypothesis testing, this guidance may not be considered appropriate, since pilot trials should not be designed to test efficacy [9–11]. Given this dearth of guidance around sample size for pilot cRCTs, studies may justify their sample size on the basis of gathering sufficient data to calculate the sample size for a main trial. In particular, since the estimated parameters such as the ICC from a pilot cRCT may be used to help design the main trial, their quantification could be used as a rationale to justify the sample size chosen for a pilot trial. However, it has been shown that for pilot cRCTs, these parameters are often so imprecisely estimated as to be uninformative [13]. For this reason, it has been recommended that an estimate of the ICC from an individual pilot study should not be the only source of an ICC estimate for main trial design [1, 13]; for example, it could be taken from other published work [14]. It is currently unclear, however, whether trialists are following this guidance, and if so, whether this has impacted on the chosen sample size and/or the given justification for that choice.

## Aim

Given the uncertainty around justifications for sample size for cCRTs, we aimed to provide an overview of the landscape of sample size cCRT studies as a starting point for further research to be conducted on what is an appropriate sample size and justification. We aimed to conduct a review to understand the choices and justifications of pilot cRCT sample sizes, and their trends over time, and to explore apparent changes in these trends following the publication of CONSORT extensions for cluster trials and for pilot and feasibility trials. We also wished to understand whether there is systematic variation in sample size choice across trial area and cluster type. We were primarily interested in the number of clusters per arm due to the significant impact this choice may have for attaining the aims of the study [13], but also explored the number of participants.

## Methods

### Search strategy

A search of PubMed and Web of Science for pilot cRCTs was undertaken. The search was carried out on 01/10/2020 and was restricted to papers published on or after 01/01/2010. This start date was chosen due to the very small number of studies per year returned using these criteria before this date. Consistent with previous research [4, 15, 16], the identification of papers was based around a search for the terms ‘pilot’ or ‘feasibility’ in the title and abstract (PubMed) or the title and topic (Web of Science). This search was supplemented with terms to identify cluster-randomised trials, giving four unique searches for each database. The full search strategy is outlined in Fig. 7 in Appendix 1).

### Date restriction and impact of COVID-19

No specific end date was set to the search, which therefore returned results up until the search date, which was in October 2020. However, this was sufficiently far into the COVID-19 pandemic that it was plausible that sample sizes for some returned records may have been impacted. It was determined that protocols received by a journal before the end of March 2020, or with a corresponding trial registration created before March 2020, were eligible for inclusion. Results papers were included when the study completed recruitment before March 2020 or could be reasonably assumed to have done so (e.g. one study was completed in April 2020 but included a 12 week follow-up period, so recruitment would have been completed before March 2020). Papers from 2020 that could not be confidently assumed to have been planned or completed enrolment before this time were excluded.

### Inclusion criteria

Since many small studies tend to be described as pilot trials when they have not been designed with a future main trial in mind [2, 4], the study being described as a pilot or feasibility study in the title was not sufficient for inclusion in this review. To be classed as a pilot or feasibility study, the abstract had to refer to at least one of the following:

- Gathering information to help with designing a future main trial;
- Assessing the feasibility of a future main trial;
- Emphasis on the collection of feasibility outcomes necessary for planning a future main trial.

We were interested in both planned and enrolled sample size, so we did not exclude any particular type of record from our search (e.g. protocols), as similar research has done [9].

### Exclusion criteria

Records were excluded from further analysis for any of the following reasons:

- Record was an abstract only (not full record);
- Not a trial;
- Not a pilot/feasibility study;
- Assessing feasibility of intervention rather than trial;
- Not controlled;
- Not randomised;
- Not cluster-randomised;
- Internal pilot/phase of larger main study;
- Not piloting full trial (just component);
- Stepped wedge/multiple crossover/factorial design;
- Substudy of pilot trial.

### Data extraction

The primary interest in the review was in the sample size of these trials, so we focused primarily on collecting both planned and enrolled sample sizes, both in terms of the number of clusters and number of participants, as well as the number of study arms. For completeness, we also collected the number of clusters and participants remaining at follow-up. In addition, information that may have had an impact on the sample size chosen was obtained, including the following:

- The therapeutic area of the study;
- The funding type;
- The cluster type;
- The participant group.

Since pilot and feasibility trials should not be conducted with the aim of hypothesis testing, which has been emphasised and made explicit by CONSORT guidelines around the conduct of such trials [9], we were also interested in the context around pilot sample sizes. We therefore collected the following relevant metrics:

- Any justification given for the chosen sample size (where more than one was given, the first two were recorded);
- Whether assessing trial feasibility was stated as the main aim;
- Whether a formal power calculation had been performed;
- Whether formal analysis had been carried out on non-feasibility outcomes.

Wherever only a protocol, or only a results paper, was returned from our original search, additional searches of the grey literature, including trial registrations, were undertaken to attempt to complete the record. Where multiple records were returned for a single study, we took the date for that study from the earliest publication or trial registration.

All papers were screened by one reviewer and another reviewer screened a sample of 50%. Disagreements were resolved by discussion with the lead reviewer or with a third reviewer when needed. As this was an audit rather than a systematic review, we did not conduct an assessment of agreement. All authors were involved in the data extraction and conducted the extraction in independent pairs.

### Analysis

Analyses were descriptive or displayed graphically. For overall sample sizes, we calculated means and standard deviations (SDs) as well as medians, interquartile ranges (IQRs), and ranges. As we anticipated highly skewed distributions of sample size, for further analyses including breakdowns of sample size by various trial features, we focused on the median, IQR, and range.

### Public and patient involvement

Patients and/or the public were not involved in the design, conduct, reporting, or dissemination plans of this research.

## Results

**Fig. 1:**
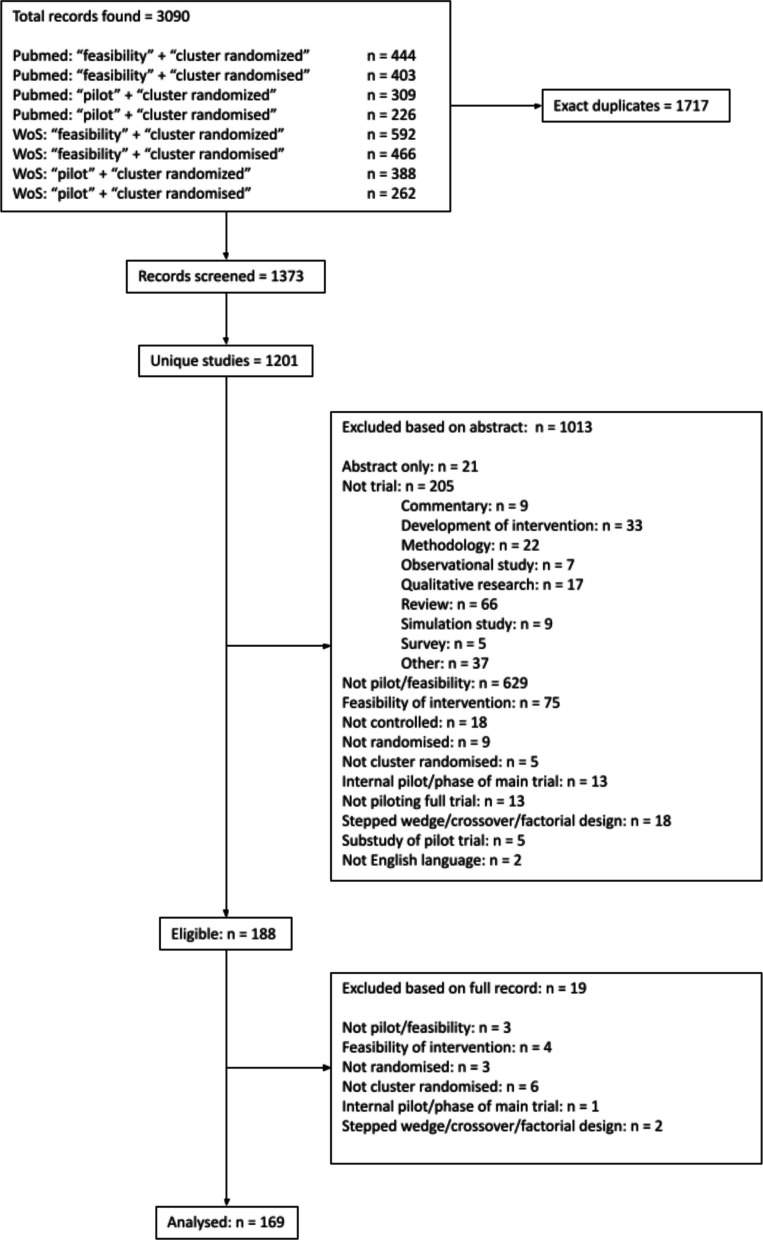
PRISMA flowchart

Our search returned 3090 individual records. After removing exact duplicates, aggregating records into unique studies (e.g. a protocol and results paper were often returned for a single study), and exclusions, we identified 170 pilot or feasibility cRCTs within 169 studies (one paper reported two distinct trials) (see PRISMA flowchart in Fig. 1).

**Fig. 2:**
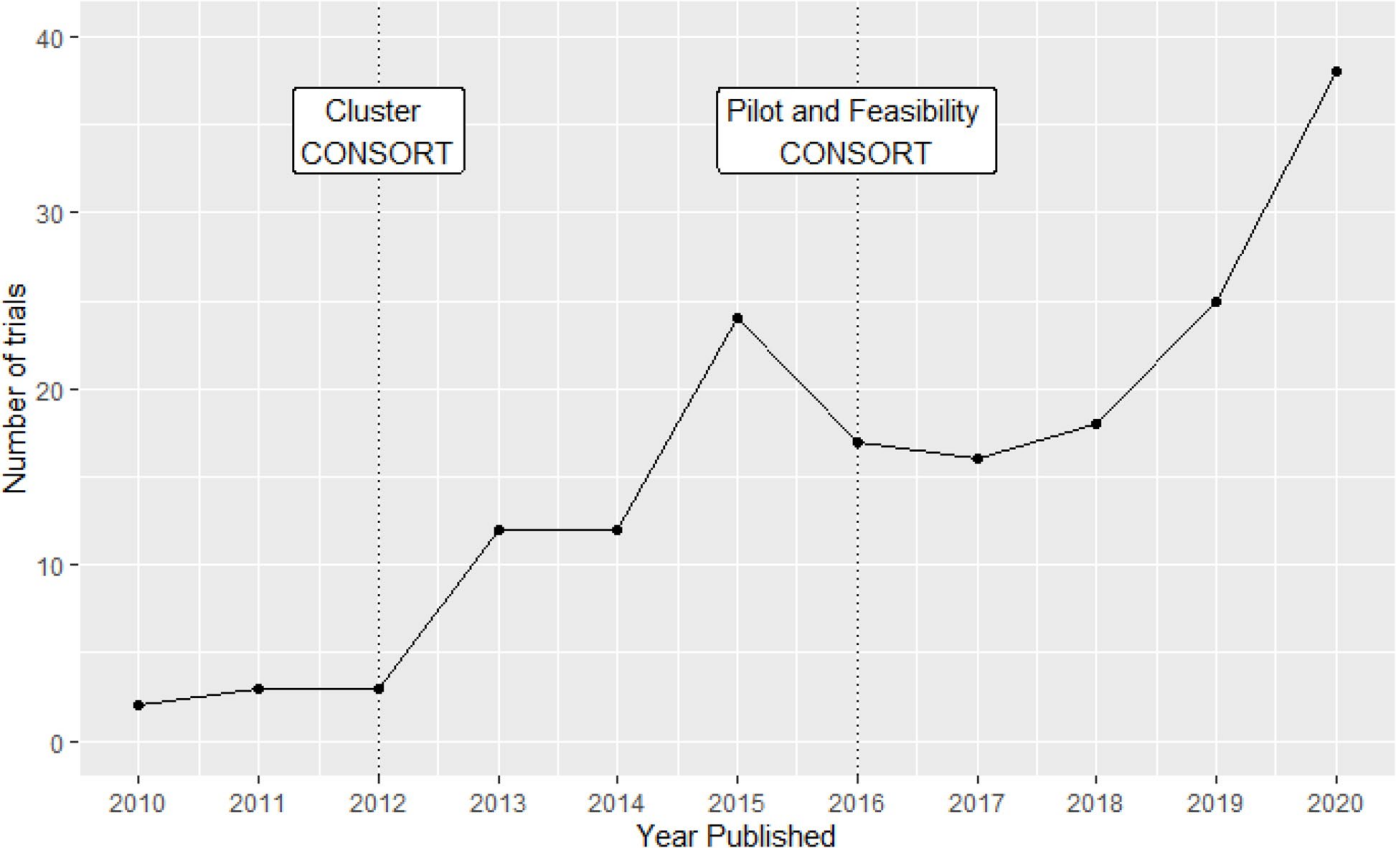
Number of pilot and feasibility trials over time

Figure 2 shows the number of pilot and feasibility trials each year over time. Following the cluster RCT CONSORT extension in 2012, there is an increase in the number of trials being published. Following the pilot and feasibility CONSORT extension in 2016, there was a continually increasing number following a slight dip.

### Study characteristics

A summary of the main characteristics of the trials included in the final review is shown in Table 1. Most studies had two arms and only two studies had more than three arms. Most studies took place in educational, primary care, and hospital settings. Notably, most studies examined behavioural interventions, particularly in the areas of health promotion and risk reduction, and there were only two drug trials. Most studies were publicly funded. Few studies were published before 2013, after which point, the number of studies published per year more than trebled. This increase occurred following the 2012 update to the CONSORT extension for cluster trials. In general, the number of studies per year continued to increase during the timespan of the review.

**Table 1:** Study characteristics

	***N***	Percent		***N***	Percent
*N* arms			Cluster type		
2	151	88.8	Care homes	14	8.2
3	17	10	Dental	1	0.6
4	2	1.2	Education	37	21.8
Year published			Geographical area	11	6.5
2010	2	1.2	Health professional	15	8.8
2011	3	1.8	Hospital	19	11.2
2012	3	1.8	Maternal services	3	1.8
2013	12	7.1	Mental health services	6	3.5
2014	12	7.1	Mixed	1	0.6
2015	24	14.1	Other	13	7.6
2016	17	10	Outpatient care	11	6.5
2017	16	9.4	Pharmacies	6	3.5
2018	18	10.6	Physiotherapy department	2	1.2
2019	25	14.7	Primary care	29	17.1
2020	38	22.4	Workplace	2	1.2
Funder type			General medical area		
Charity	9	5.3	Condition management	25	14.7
Mixed	22	12.9	Health promotion	28	16.5
Not stated	4	2.4	Mental health	16	9.4
Private	7	4.1	Other	5	2.9
Professional group	4	2.4	Prevention	21	12.4
Public	124	72.9	Quality of care	32	18.8
Drug trial			Quality of life	11	6.5
*N*	168	98.8	Risk reduction	22	12.9
*Y*	2	1.2	Sport and exercise medicine (SEM)	10	5.9
Total	170	100	Total	170	100

### Sample size

Summaries of the sample sizes are given in Table 2. For planned, enrolled, and end of study, the median number of clusters was four per arm.

**Table 2:** Summary of sample sizes

	***N***	Mean	SD	Median	IQR	Range
Total clusters						
Planned	143	13.9	23.0	8.0	6.0–12.0	2–200
Enrolled	145	14.4	21.7	8.0	6.0–12.0	2–184
End of study	138	12.2	17.6	8.0	6.0–12.0	2–162
Clusters per-arm						
Planned	143	6.5	10.6	4.0	2.8–6.0	1–100
Enrolled	145	6.7	8.9	4.0	3.0–6.0	1–61.3
End of study	138	5.7	7.3	4.0	2.3–5.5	0.7–54
Total participants						
Planned	147	311.1	439.5	150.0	80.0–360.0	20–3000
Enrolled	146	332.8	617.9	136.5	65.0–296.8	10–4236
End of study	142	265.1	524.9	104.0	56.25–228.25	8–4236
Participants per-arm						
Planned	147	148.3	215.1	70	39.5–180.0	10–1500
Enrolled	146	155.7	278.7	67	30.3–145.1	3.3–1820
End of study	142	122.4	224.2	51.25	24.8–112.3	3–1412
Participants per-cluster						
Planned	134	41.7	75.4	16.7	10.0–35.3	1.2–500
Enrolled	144	39.4	75.0	15.6	8.8–36.3	0.6–606.7
End of study	134	32.6	59.1	12.8	6.6–32.9	0.5–435.2

### Planned sample size by year

**Fig. 3:**
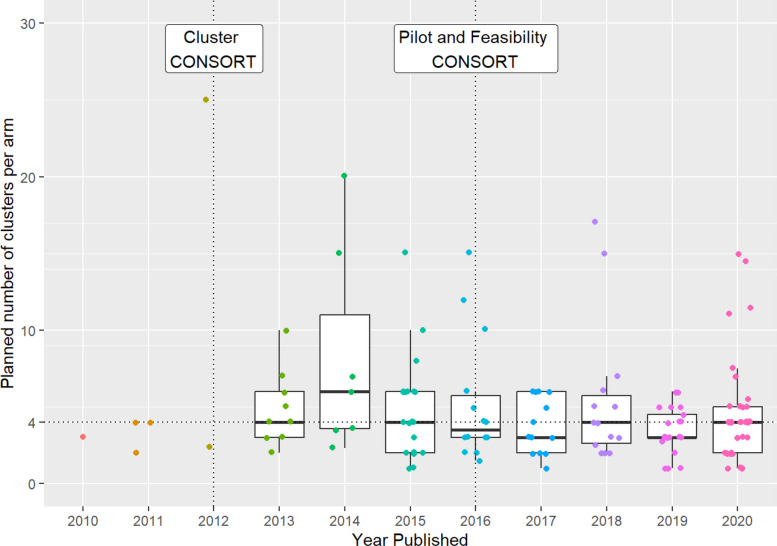
Boxplot showing the distribution of planned clusters per arm for each year of the study

Median sample size was typically around four clusters per arm for each year of the study (Fig. 3). Whilst there was considerable variation amongst individual studies, the interquartile range of the sample size was also fairly consistent across years with a large number of studies (2015 onwards). The median sample size does not appear to have been affected by the publication of CONSORT extensions.

### Sample size and study aims

**Fig. 4:**
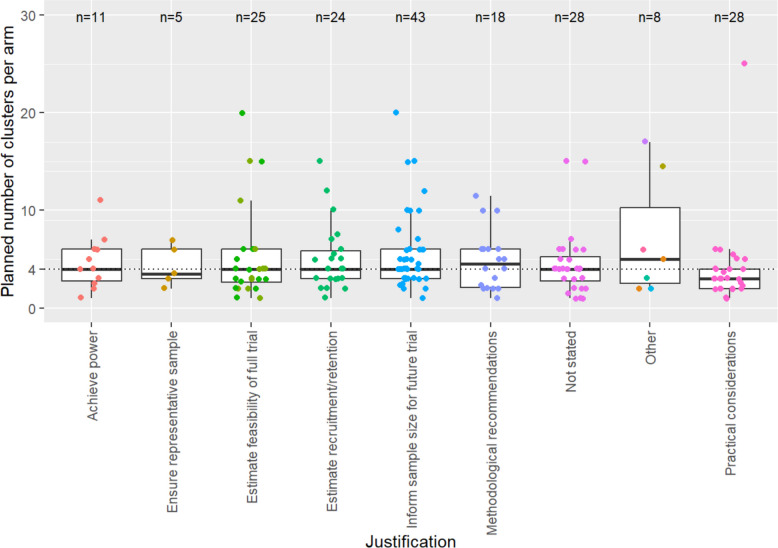
Boxplot showing the distribution of planned clusters per arm by justification type. Note: ‘Methodological recommendations’ were situations where the authors cited a paper with a recommendation for the sample size such as reference 12; ‘Practical considerations’ included any papers which stated sample sizes for practical reasons such as costs and time; ‘Other’ was a group made up of the following: Achieve qualitative data, Estimate contamination rate, Estimate response rates, Fixed percent of necessary sample for full trial, Inform delivery of intervention, Non-specific precision calculation, and Safety

Figure 4 illustrates the planned sample size according to the justification given for the choice of sample size. Regardless of the justification, the median sample size is again around four, with similar levels of variation indicated by the IQR. Table 3 gives the planned sample size according to other features of the study. This indicates that the median sample size is not significantly impacted by whether the main aim of the study was stated as feasibility, whether a formal power calculation was performed to determine the sample size, whether a formal analysis was carried out, or whether the ICC was estimated. Further boxplots broken down by different study characteristics can be found in Fig. 8 in Appendix 2 which present similar results.

**Table 3:** Planned number of clusters per arm by key features of trial

	***N***	Mean	SD	Median	IQR	Range
Main aim stated as feasibility						
Yes/planned	129	6.8	11.1	4	2.7–6	1–100
No	14	4.1	3.33	3	3–4	1.5–15
Formal power calculation performed						
Yes/planned	30	6.2	7.5	4	2.1–6.8	1–37
No	113	6.6	11.3	4	3–6	1–100
Formal analysis on non-feasibility outcome						
Yes/planned	54	8.0	14.7	4	3–6	1–100
No	89	5.6	6.7	4	2–5	1–41
ICC estimate reported						
Yes/planned	58	6.0	7.1	4	3–6	1–50
No	85	6.9	12.4	4	2–6	1–100

**Fig. 5:**
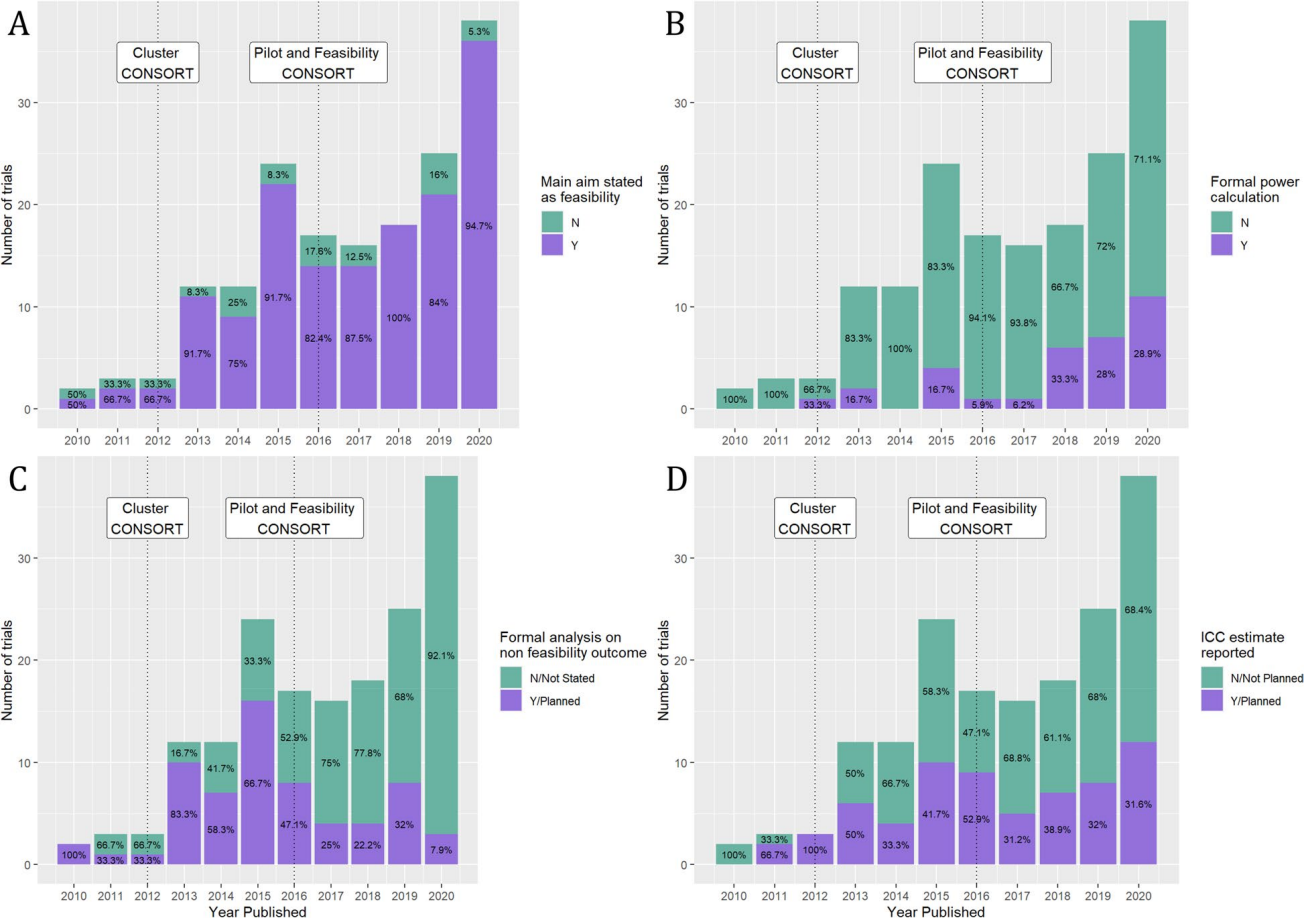
Bar charts of study key features over time: **A** The main aim was stated as feasibility. **B** Formal power calculation. **C** Formal analysis on a non-feasibility outcome. **D** ICC estimate reported

The four plots in Fig. 5 show the frequency of stated aims of pilot cRCTs over time. Figure 5A shows the number of trials over time, which stated their main aim as feasibility. The proportion stating their main aim as feasibility increases slightly over time but is a consistently high proportion. Figure 5B shows the number and proportion of trials over time which had a formal power calculation, indicating that the number of studies conducting a power calculation has increased. Figure 5C shows the number of trials over time which conducted a formal analysis on a non-feasibility outcome (e.g. clinical outcome). This suggests that the proportion conducting formal analysis is generally decreasing over time. Figure 5D shows the number of trials over time who planned to or reported an ICC estimate, indicating a marginal decrease in the proportion reporting or planning to report the ICC estimate, although this trend is not strong.

**Fig. 6:**
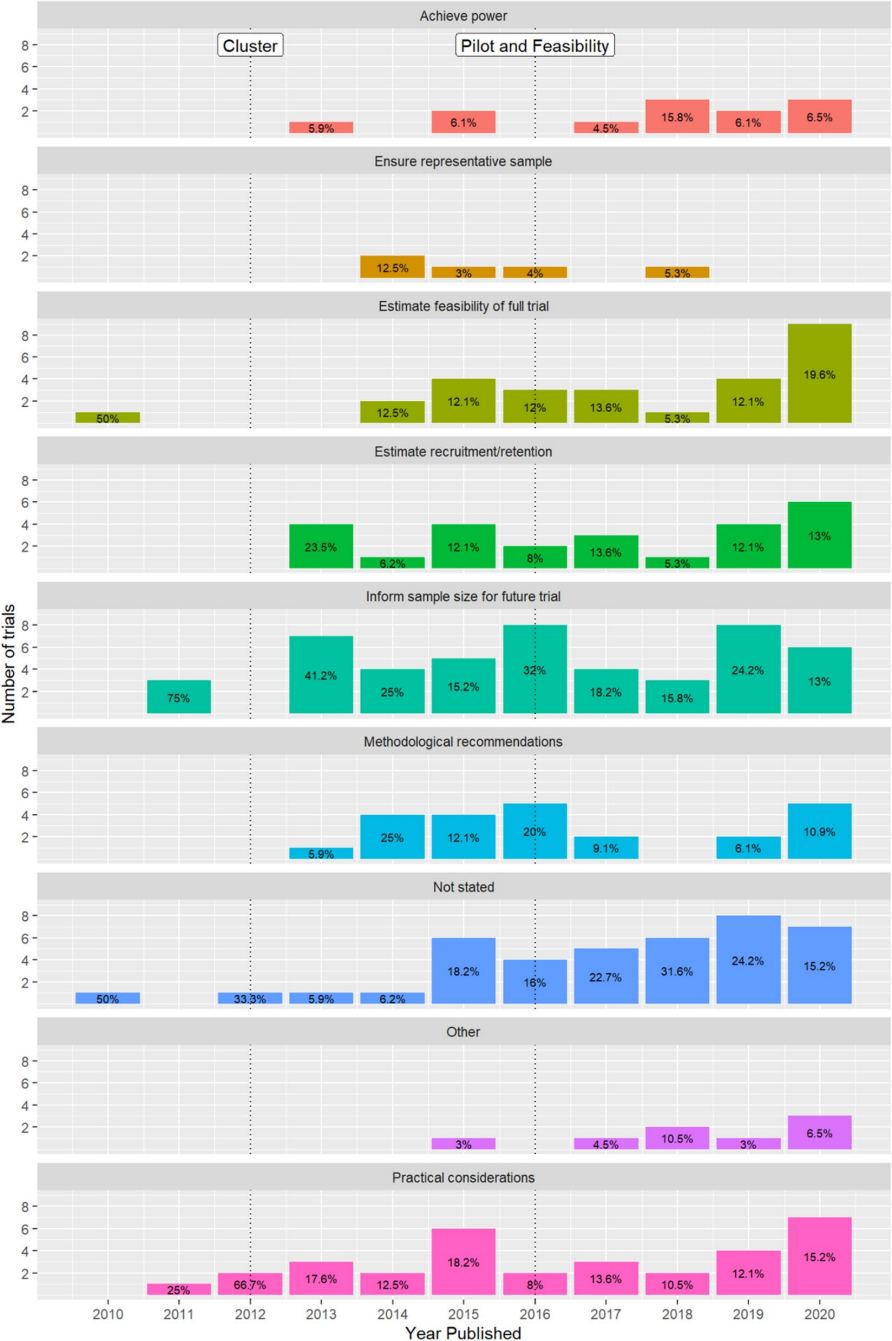
Sample size justification over time

**Fig. 7:**
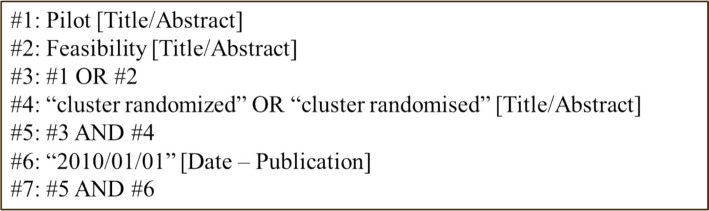
Search terms

**Fig. 8:**
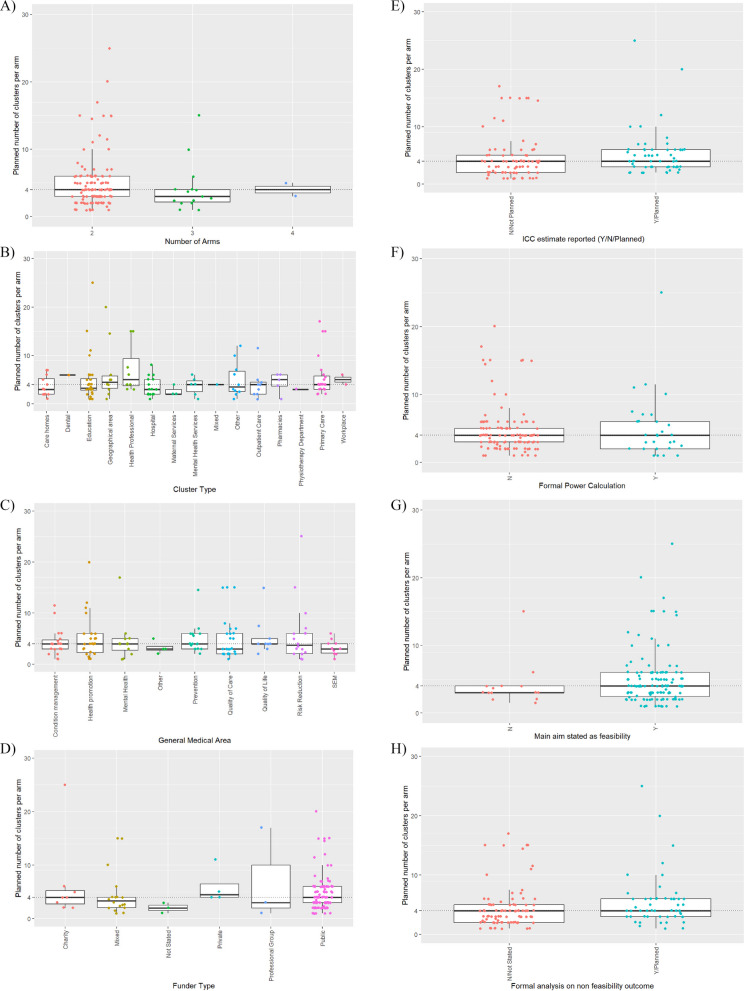
Box plots over time for all other collected variables. **A** Number of arms. **B** Cluster type. **C** General medical area. **D** Funder type. **E** ICC estimate reported. **F** Formal power calculation. **G** Main aim stated as feasibility. **H** Formal analysis on non-feasibility outcome

Figure 6 shows the number of studies over time reporting different sample size justifications. For most types of justification, the number of studies citing them has increased over time. The most common justification was ‘to inform sample size for a future trial’ (21.82% overall) and following that, ‘not stated’ accounted for 18.2% overall.

## Discussion

We found that the median sample size from 170 pilot cRCTs, published from 2010 up until the COVID-19 pandemic, was consistently around four clusters per arm. This was broadly consistent regardless of the justification given for the sample size, whether a formal analysis was planned, and whether the intention was to estimate the ICC. The lowest median planned clusters per arm was for trials basing sample size on practical considerations, which is possibly not unexpected given the justification. The consistency across all study aims and justifications was unexpected, since the number of clusters employed in a pilot cRCT has significant implications for meeting various study aims.

The median sample size was also consistent across cluster type, general medical area, and funding type, as well as over time. From 2015, even the interquartile range of the sample size is fairly consistent, suggesting more consistent trends over more recent years. There was a notable increase in the number of studies published from 2013, which may have been a result of the 2012 CONSORT extension for cluster trials. However, other outcomes did not appear to be affected by guidance produced in either the 2012 CONSORT extension for cluster trials or the 2016 extension for pilot trials. In comparison, a systematic review published in 2022 conducted between MEDLINE’s inception and December 2020 looking at feasibility cRCTs in schools found results that mirrored the findings in this paper. The median total number of clusters in the study was 7.5 (with 21/24 having two arms), which is consistent with median estimate of four per arm found in this review [17].

The sample size of four per arm may be an appropriate choice under certain circumstances. For example, Lewis and Julious [18] showed that when estimating a sample size for a main trial with a continuous outcome, which would account for the imprecision in the ICC estimate, the greater the number of clusters in the pilot trial estimating the ICC, the greater the precision, and so the smaller the final (main trial) sample size. They showed that for cluster sizes of 15–20 participants, the main-trial sample size asymptotes for ICC estimates generated using 4–8 clusters per arm in the pilot trial. This implies that the *minimum* sample size for a pilot cRCT should be four clusters per arm but may be larger depending on the specific aims and parameters of the study. Four clusters per arm is also consistent with the one piece of explicit guidance available for sample size for pilot cRCTs [12]. Some studies cited this guidance in their sample size justification, although none referred to the methodological basis of this guidance: that this is the smallest sample size necessary for a *t*-test or Wilcoxon test. For studies not planning to use such tests, it is not clear that this guidance in of itself would be appropriate.

### Influences on choice of sample size

The proportion of studies stating their main aim as assessing feasibility of a main trial has increased marginally over time, in line with best practice guidelines [9]. However, this trend is not a strong one. Notably, whilst the proportion of trials performing a formal power analysis does not appear to show an obvious decreasing trend, the proportion performing formal analysis (i.e. hypothesis testing) does appear to have decreased over time, again in line with good practice. The reasons for this inconsistency are not clear.

The proportion of trials reporting (or planning to report) the estimated ICC has also slightly decreased. This may be a consequence of guidance suggesting that estimates of this quantity from pilot trials are usually extremely inaccurate [13]. However, whilst an isolated specific point estimate of the ICC from a pilot study may be too imprecise to utilise in a main trial sample size calculation, it may still be useful to report the ICC from pilot studies in order to contribute to larger bodies of evidence regarding typical ICCs for certain cluster types [19–21].

Justifications for the choice of sample size do not appear to have changed notably over time, although ‘informing main trial sample size’ appears to have marginally decreased, and ‘estimating feasibility of full trial’ slightly increased. These too may reflect best practice guidelines. However, despite recommendations that pilot studies should justify their choice of sample size, the proportion of studies in this review that provided no justification remained high even in the later years (15–25%), consistent with previous work showing poor rates of reporting sample size justification in pilot cRCTs [15].

Whilst there have been some minor changes over time in the apparent intention and practice of pilot cRCTs, some of which appear to be in line with guidance, there has not been a corresponding change in the choice of sample size, which has remained consistently around four clusters per arm. No overall aim or sample size justification appeared to materially impact the actual choice of sample size. This raises a question around how influential the stated aims and justifications actually are around the choice of sample size, and how informative the stated justifications are, in particular.

Given that aims and justifications appear to have changed, but typical sample sizes have not, it is possible that some researchers choose sample size for pilot cRCTs primarily out of convenience or practical reasons, but that there is a perceived need to provide different justifications that may be based in statistical theory or more directly connected to the stated aims of the study. The present paper thus points to the influence and potential drawbacks of broad and general sample size recommendations. Investigators are under many pressures, including ethical and financial, to make their pilot studies as small as possible [22]. Ethical pressures exist because pilot studies are unable to decide on efficacy of treatment, and so it is important to minimise the number of people that will receive no direct benefit from being in a study. Financial pressures exist because it is challenging to acquire funding for pilot studies, and cluster pilots will be larger than individually randomised studies. As a result, investigators may rely on any published guidance that exists for justification of a small sample, even if not relevant to the aims of the study. However, the only current published guideline is based on a test of efficacy, which should not be an aim of a pilot study. Our findings lead us to suggest that investigators should carefully consider the aims and objectives of their pilot study and ensure that the planned sample size will be sufficient to meet these and avoid relying on generic sample size recommendations simply to provide a justification which may not be relevant or appropriate for their situation.

## Future research

This paper is limited by the pre-COVID-19 end date to the review. This end date was chosen for practical considerations in order to avoid any artefactual differences in pilot cRCT design and sample size choice that may have resulted from the pandemic itself, as opposed to changes in trends that may result from improving practice. It is however important to understand whether trends have altered since the pandemic, and what any changes in more recent years may look like.

This paper only describes trends in sample size for pilot cRCTs and does not address whether these trends are appropriate and sufficiently meet the needs and aims of such studies. Future research should aim to establish whether four clusters per arm is sufficient for the typical justifications given by the papers included here, and if not, what an acceptable sample size might be for given sets of conditions. Hemming et al. have written a tutorial paper and developed a shiny app which can be used to help determine the sample size for external pilot cRCT [23].

It would also be pertinent for future research to address the question of how closely pilot cRCTs should mimic the intended main trial in terms of their design, which would provide a better contextual landscape in which to restrain pilot cRCT design and in particular the choice of and justification for their sample size.

## Conclusion

Average sample sizes for cRCTs have remained strikingly constant over the period 2010–2020 and across several key features of studies and do not appear to be meaningfully impacted by the stated study aims or sample size justifications. This is despite the fact that the reported main aims for pilot cRCTs, and justifications for their sample sizes, do appear to have changed during this time. In general, these changes in aims and justifications reflect best practice and may generally be considered improvements to the design of these trials. Whilst we did not see any clear relationship between these changes and the relevant CONSORT extensions, it is likely that they have influenced these changes, along with guidance around the inaccuracy of estimated ICCs.

## Supplementary Information

Additional file 1: PRISMA 2020 Checklist.
